# Evidence for the presence of African swine fever virus in an endemic region of Western Kenya in the absence of any reported outbreak

**DOI:** 10.1186/s12917-016-0830-5

**Published:** 2016-09-08

**Authors:** Lian F. Thomas, Richard P. Bishop, Cynthia Onzere, Michael T. Mcintosh, Karissa A. Lemire, William A. de Glanville, E. Anne J. Cook, Eric M. Fèvre

**Affiliations:** 1 Centre for Infection Immunity, and Evolution, Institute for Immunology and Infection Research, School of Biological Sciences, University of Edinburgh, Ashworth Labs, West Mains Rd, Edinburgh, EH9 3JT UK; 2 International Livestock Research Institute, PO Box 30709, Nairobi, 00100 Kenya; 3 United States Department of Agriculture, Foreign Animal Disease Diagnostic Laboratory, National Veterinary Services Laboratories, Animal and Plant Health Inspection Services, PO Box 848, Greenport, NY 1944 USA; 4 Institute for Infection and Global Health, University of Liverpool, Leahurst Campus, Chester High Road, Neston, CH64 7TE UK

**Keywords:** African swine fever virus, Epidemiology, Kenya, Slaughter house, p72 PCR, ASFV real time PCR, Genotype IX

## Abstract

**Background:**

African swine fever (ASF), caused by African swine fever virus (ASFV), is a severe haemorrhagic disease of pigs, outbreaks of which can have a devastating impact upon commercial and small-holder pig production. Pig production in western Kenya is characterised by low-input, free-range systems practised by poor farmers keeping between two and ten pigs. These farmers are particularly vulnerable to the catastrophic loss of livestock assets experienced in an ASF outbreak. This study wished to expand our understanding of ASFV epidemiology during a period when no outbreaks were reported.

**Results:**

Two hundred and seventy six whole blood samples were analysed using two independent conventional and real time PCR assays to detect ASFV. Despite no recorded outbreak of clinical ASF during this time, virus was detected in 90/277 samples analysed by conventional PCR and 142/209 samples analysed by qPCR. Genotyping of a sub-set of these samples indicated that the viruses associated with the positive samples were classified within genotype IX and that these strains were therefore genetically similar to the virus associated with the 2006/2007 ASF outbreaks in Kenya.

**Conclusion:**

The detection of ASFV viral DNA in a relatively high number of pigs delivered for slaughter during a period with no reported outbreaks provides support for two hypotheses, which are not mutually exclusive: (1) that virus prevalence may be over-estimated by slaughter-slab sampling, relative to that prevailing in the wider pig population; (2) that sub-clinical, chronically infected or recovered pigs may be responsible for persistence of the virus in endemic areas.

**Electronic supplementary material:**

The online version of this article (doi:10.1186/s12917-016-0830-5) contains supplementary material, which is available to authorized users.

## Background

African swine fever virus (ASFV) is a highly infectious virus of the family *Asfarviridae* and is the causative agent of African swine fever (ASF) infection which may lead to mortality of up to 100 % in naïve domestic pig populations [1]. Per-acute and acute ASF present as high fever, anorexia, depression, cutaneous hyperaemia (per-acute), erythema and or areas of cutaneous cyanosis particularly of the abdomen, ears and distal extremities with sudden death within 1–7 days [2]. Low virulence strains of the virus have been reported and there is evidence from Senegal, Cameroon, Nigeria, Madagascar, Malawi, Mozambique, Zambia and possibly Angola that pigs in endemic areas may have developed resistance or that chronic or sub-clinical infections may be increasing [3–6].

ASFV has at least three distinct transmission cycles; (1) involving an argasid soft tick (genus *Ornithodoros*) and warthogs; (2) between argasid tick vector and domestic pigs and (3) direct pig to pig transmission without the tick vector. Direct pig to pig transmission is probably the major mode of transmission across Africa in areas not adjacent to national parks [7, 8]. The role of other wild suiforms (bush pigs, wild boars and giant forest hogs) in the epidemiology of ASFV is yet to be fully understood [8]. Pig to pig transmission can occur both through contact between live pigs, fomites or ingestion of infected pork meat [4].

Pig keeping in Kenya generally occurs as a small-holder enterprise with between two and ten pigs per farm [9, 10]. The majority of pigs are kept under a free-range system [11], especially in urban and peri-urban areas [12]. In western Kenya where this study was conducted pigs kept under a free-range system travel an average of 4 km very 12 h whilst scavenging for food within a mean home range of 10,343 m^2^ (range 2937–32,759 m^2^) [13]. This scavenging behaviour puts these pigs at risk of acquiring multiple infectious organisms, including ASFV from the environment, neighbouring domestic pigs, or wildlife reservoirs.

There is currently no vaccine or chemotherapeutic available for ASFV and control therefore relies on preventing contact of pigs with the virus. Free-range pig production systems, as utilised in Kenya, require considerable improvements to biosecurity in order to prevent pigs coming into contact with potentially infected animals or animal products [4].

Several outbreaks of ASF have been reported in western Kenya. Prior to the instigation of this study there were two outbreaks between October 2006 and February 2007 resulting in 82 porcine deaths from Busia (formerly Western Province) and Kisumu (formerly Nyanza Province) [14], and after the completion of the present study, between December 2010 and March 2012, 163 porcine deaths were reported in Mahiakolo (formerly Western Province) and Kisumu East (formerly Nyanza Province) [15]. Outbreaks of diseases causing high mortality, such as ASF, are of high concern to poor farmers such as those in western Kenya, with the potential to catastrophically threaten their livelihoods through the rapid loss of their livestock assets [16].

Exactly how the virus persists within endemic pig populations is not known but a role has been proposed for survivor, sub-clinical and chronically infected pigs to maintain virus [17]. There is evidence that recovered animals can transmit the virus to naïve populations for up to 3 months [18] and may be persistently infected with virus for up to 6 months [18, 19]. Low virulence strains of ASFV have been reported in the Dominican Republic, Spain and Portugal, which appear to lead to chronic infections with prolonged viremia lasting several weeks to several months [20]. These chronic infections, however, have never been reported in the African continent [2].

Viral DNA has been detected in asymptomatic pigs in both south-western Kenya [21], Uganda [22] and Tanzania [23]. Genotyping suggests that the isolates associated with this situation in Kenya are likely of low virulence (Genotype X) and of high virulence in Tanzania (Genotype II- Georgia 2007). In Mozambique, populations of pigs have been identified with high levels of circulating antibodies, suggesting resistance to virulent isolates [6]. Reasons for the apparent resistance of some pigs to potentially virulent isolates of ASFV have been suggested but are as yet unproven. Breed resistance has been proposed [23] but heritability did not appear to be the case in Mozambique with all offspring of apparently resistant pigs succumbing to challenge by isolates of the same genotype [6].

As western Kenya is an area where ASF is a regular occurrence, an ongoing programme of porcine sampling presented and opportunity to further investigate the epidemiology of ASFV during a period when no outbreaks were reported. This study therefore aimed to investigate the presence of ASFV viral DNA in domestic pigs presented to slaughter during a time period with no officially reported outbreaks of ASF and, should any virus be found, to determine which genotype was represented.

## Method

### Study site

**Fig. 1:**
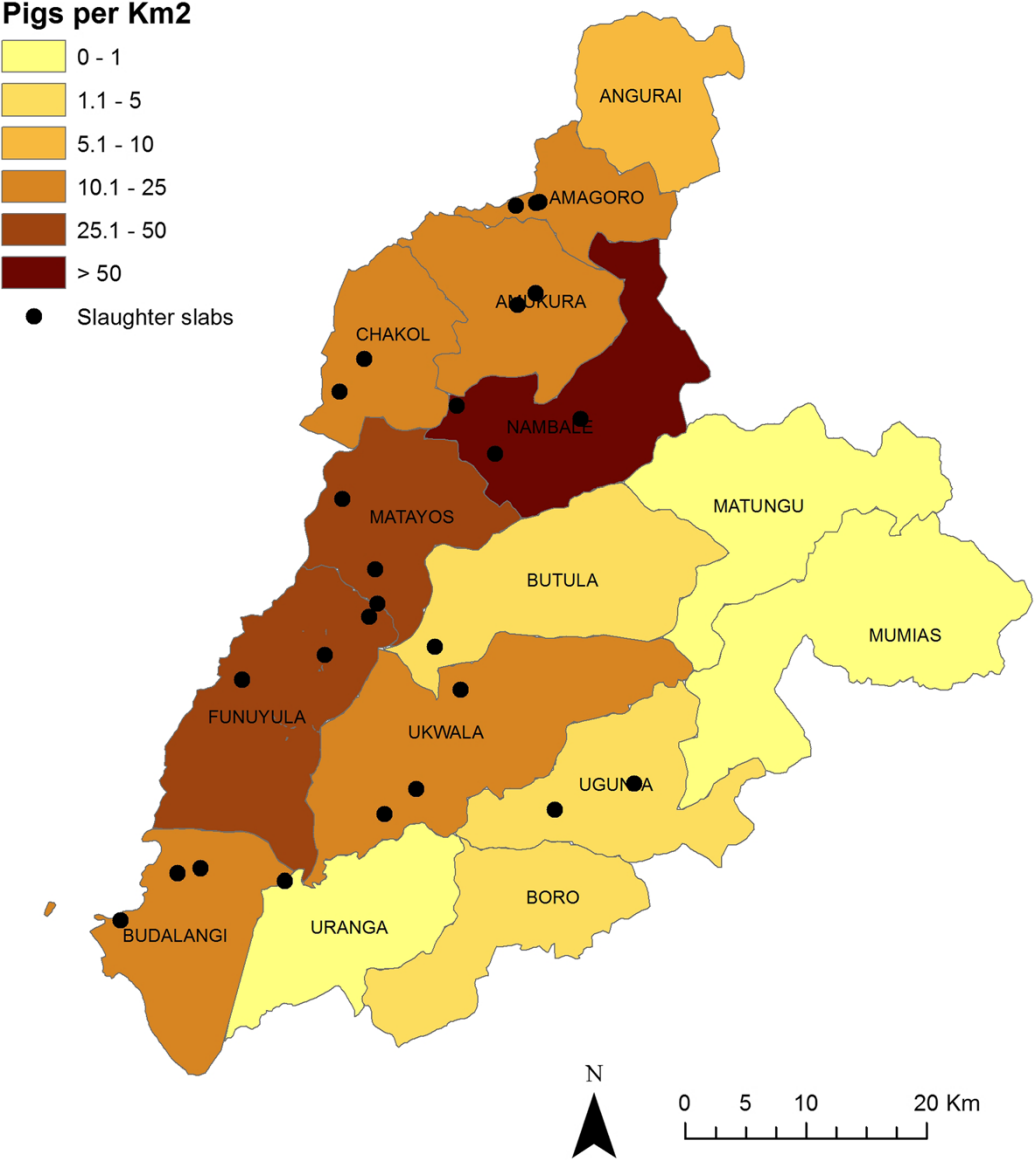
Map depicting study site showing divisional pig population density and location of registered porcine slaughter facilities at time of sampling. This map was produced using ArcMap^TM^ version 9.1 with geographical data provided by ILRI GIS unit http://www.ilri.org/gis and pig population data provided by the District Livestock and Production Office 2009 figures and overlaid with the location of slaughter facilities collected in the field using a hand held Garmin® eTrex GPS unit

This study represents a sub-study of the “People, Animals & their Zoonoses” (PAZ) project in western Kenya [24]. The study site is a 45 km radius semi-circle from Busia Town and is largely representative of the Lake Victoria Crescent ecosystem. It is bounded to the west by the border with Uganda, to the south by Lake Victoria and to the North by Mount Elgon. The ten divisions (the 3^rd^ largest administrative unit prior to the 2013 constitution reform) with the highest pig-population and at least one registered porcine slaughter facility (slab) were purposively selected from within the study site; each selected division contained over 2 % of the total pig population of the study area. Figure 1 illustrates the larger ‘PAZ’ study area, the pig population density (according to the District Livestock and Production Office 2009 figures) and the location of registered slabs.

### Sample collection

Sample collection took place at slabs in the selected divisions, which were registered at the time of sampling with the District Veterinary Office. The pathogen of primary focus for this sampling was *Taenia solium* as previously described [25] and the sample size (319) was calculated accordingly, using WinEpiscope 2.0 [26] with an assumed *T. solium* prevalence of 14 % [12] with 5 % precision and 99 % confidence level. Our interest in ASFV was solidified after the sampling frame was devised at which point a collaboration was brokered which enabled testing of these samples, this unfortunately resulted in a lack of appropriate samples being obtained from the first 66 pigs sampled.

Twenty-six registered slabs were identified and were visited on the day of highest through-put, as identified by the meat inspector or slab owner, and all pigs being slaughtered on the day of visit were sampled. In these low-throughput slaughter slabs producing pork for local consumption, categorised as ‘C’ by the Kenya Meat Control Act generally 1–4 pigs are slaughtered per day, with a legal maximum of 6 pigs slaughtered on any one day [27]. Facilities were re-visited until a quota proportional to the percentage of the total pig population in that division had been fulfilled as indicated in Table 1.

**Table 1:** Divisional pig population, sampling quota and number of registered porcine slaughter facilities at the time of sampling

Division	Pig population	% of total study site pig population	Quota of pigs to sample	No. of pigs sampled	No. of registered slabs
Amagoro	1418	3.2	10	10	3
Amakura	3800	8.5	27	21	5
Budalangi	2640	5.9	19	19	4
Butula	1010	2.3	7	13	2
Chakol	1950	4.4	14	14	2
Funyula	8910	19.9	63	53	1
Matayos	5700	12.7	41	20	2
Nambale	14680	32.8	105	103	3
Ugunja	912	2.0	6	7	2
Ukwala	3700	8.3	27	17	3
Total	44720	100	319	277	27

All sample collection was undertaken by a small research team of one veterinarian (the lead author) and two animal health assistants according to protocols devised by the lead author. All members of the team were present for every sampling event except in the occasion of illness or unavoidable travel.

The permission of the person presenting the pig for slaughter was sought prior to sampling and the division of origin of the pig was recorded. Pigs were restrained with a pig snare behind the canine teeth, a brief visual exam was conducted and anterior vena cava blood samples were collected in BD Vacutainer® 4 ml EDTA tubes [28]. Blood samples were transported to the laboratory, on ice, where they were stored at −40 °C until transported, on dry ice, to the International Livestock Research Institute (ILRI) facility in Nairobi where they were stored at −80 °C for 2 to 7 months prior to analysis. After each slab visit the government meat inspector was called and asked if any carcass or part thereof was condemned that day for any reason.

### African swine fever virus detection by conventional and real time PCR

Total DNA was extracted from each blood sample using the DNeasy Blood and Tissue kit (Qiagen, Hilden, Germany) according to the manufacturer’s instructions. Negative extraction controls (NEC) consisting of sterile phosphate buffered saline (PBS) pH 7.0 were included during DNA extraction to check for contamination. Conventional PCR was performed using the ASFV diagnostic primers PPA1 and PPA2 that target the highly conserved VP72 capsid protein coding region of the ASFV genome [29]. No template controls (NTC) were included during PCR. Positive extraction and amplification controls (PEC & PAC) consisted of a known ASFV positive DNA sample extracted from an ASFV positive spleen tissue. Positive samples were identified by the presence of a discrete band of 257 base pairs (bp) after electrophoresis through a 2 % agarose gel.

DNA samples were further tested at the United States Department of Agriculture (USDA) Foreign Animal Disease Diagnostic Laboratory in Plum Island NY using a modification of the ASFV real-time PCR (qPCR) which has a diagnostic specificity of 100 % and was therefore selected as an ideal confirmatory tool [30]. Briefly, 2.5 μl DNA was amplified in 25 μl reactions containing 0.3 μM forward and reverse ASFV primers, and 0.2 μM ASFV TaqMan®FAM^TM^-MGB probe using firstly the TaqMan® EZ-RT-PCR Kit (Applied Biosystems Foster City, CA, USA), and later repeated using the Life Technologies Path-ID Multiplex One Step PCR Kit (Thermo Fisher Scientific, Waltham, Massachusetts, USA) as the TaqMan®EZ reagents were discontinued by the manufacturer. Controls included an NEC consisting of 2.5 μl nuclease-free water, PEC and PAC consisting of 2.5 μl of DNeasy extracted DNA from the ASFV Killean III strain cultivated in primary porcine macrophages. qPCR was conducted using the Applied Biosystems 7500 fast platform (Thermo Fisher Scientific, Waltham, Massachusetts, USA) in standard mode with automatic baseline. Cycling conditions were 95 °C for 10 min denaturation and activation followed by 45 cycles of 95 °C for 10 s and 60 °C for 30 s. Positive reactions were identified when fluorescence exceeded 0.2 units prior to or at a cycle threshold (Ct) of 40. Results beyond the qPCR cut-off Ct of 40 or undetermined were considered negative or undetermined, respectively. Results with Ct < 30 were considered as strong positives by qPCR.

In order to avoid potential cross-contamination, DNA extraction was conducted in batches of 20 including extraction controls and deep cleaning of the hood and pipettes were done using 10 % hypochlorite and 70 % ethanol between the batches. All PCR reagents were manipulated separately and ahead of sample handling. PEC and PAC, derived from known ASFV positive material and assayed along with test samples, were manipulated only after test samples were processed and manipulated for PCR. All negative and positive PCR and qPCR controls performed as expected.

### Genotyping

To identify the ASFV genotype(s) in the positive samples; genotypic analysis of 20 randomly selected positive samples was carried out by analysis of two polymorphic loci. This included:

I. The 3′-variable end of the *B646L* gene that encodes the major capsid protein p72 by utilization of the p72U/p72D primer set in the amplification of a 478 bp region [31].
II. The *E183L* gene that encodes the p54 ASFV protein critical in the recruitment of envelope precursors to the assembly site [32] by amplification of a 676 bp region using the PPA89/PPA722 primer set [33].

The discrete bands were directly cut and purified from the agarose gels using the gel purification kit (Qiagen, Hilden, Germany) according to the manufacturer’s instructions. Sequencing was then carried out using the Sanger sequencing method. Conflicts within the sequence reads were identified using BIOEDIT [34] and contigs were built using CAP3 [35] available in the Mobyle portal (http://mobyle.pasteur.fr/cgi-bin/portal.py#forms::cap3). Reference sequences corresponding to the two loci were retrieved from Genbank (http://www.ncbi.nlm.nih.gov/genbank/) for comparison to the data obtained from Sanger sequencing. Multiple sequence alignments for each locus with reference to the Genbank sequences were performed using MEGA version 6.06 [36] CLUSTAL W [37] to determine the ASFV genotype(s) associated with the positive samples. The alignment data was transferred onto CLC genomics workbench for visualization of insertion or deletion of bases (INDELS) and measurement of conservation (http://www.clcbio.com/products/clc-genomics-workbench/). Phylogenetic analysis of each locus was executed using MEGA version 6.06 [36] and the evolutionary history was inferred using the Minimum Evolution (ME) method [38] after application of the Neighbor-joining algorithm in generation of the initial tree. The evolutionary distances were computed using the p-distance method and the ME trees were searched using the Close-Neighbor-Interchange (CNI) algorithm at a search level of 1. Data from both loci were resampled 1000 times using the bootstrap method [39].

### Statistical analysis

As the study described here comprised ‘convenience’ sampling, with a sample size and strategy based upon an unrelated pathogen, we did not feel it was appropriate to state the prevalence within this population.

## Results

Three hundred and forty three pigs were sampled in this survey. All animals appeared to be asymptomatic for ASF on visual examination by the animal health assistants and veterinarian conducting the sampling procedure and government meat inspectors reported no sick animals. Whole blood samples were available for DNA extraction and conventional PCR from 277 pigs, of which 90 samples were positive for ASFV. A subset (207) of the 277 samples were independently tested using a modified real time PCR at the USDA. In total 152 of 207 samples tested positive by qPCR using the Path-ID reagents, of which 23 samples represented strong positives (ct values <30) (The results of the conventional and qPCR analysis are shown in Table 2 and Additional file 1).

**Table 2:** Detection of African swine fever virus in pigs at slaughter in western Kenya detected by conventional and qPCR

Division of slaughter	No. pigs positive by conventional PCR	No. pigs positive by qPCR (Path-ID)
Amagoro	7/10	6/7
Amakura	5/21	9/17
Budalangi	4/19	11/17
Butula	2/13	2/6
Chakol	7/14	7/9
Funyula	22/53	31/40
Matayos	4/20	13/14
Nambale	30/103	64/84
Ugunja	3/7	1/4
Ukwala	6/17	8/9
Total	90/277	152/207

**Fig. 2:**
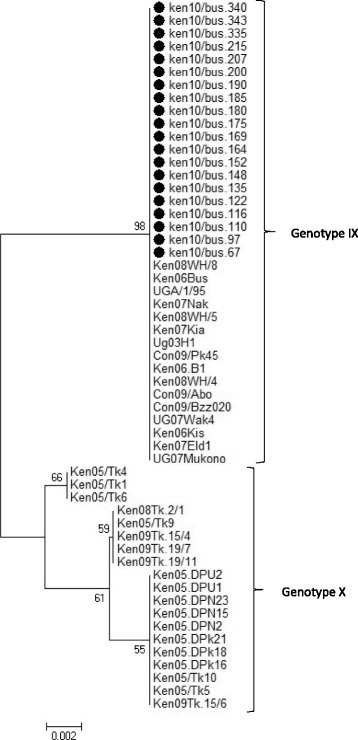
Phylogenetic tree based on the 3‘-variable end of the B646L gene.  Indicates the 20 nucleotide sequences analyzed in this study in comparison to 35 reference sequences obtained from Genbank. The 20 sequences clustered within ASFV genotype IX. The evolutionary history was inferred using the Minimum Evolution (ME) method after initial application of the Neighbor-joining algorithm. The percentage of replicate trees in which the associated taxa clustered together in the bootstrap test (1000 replicates) are shown next to the branches. The tree was drawn to scale, with branch lengths in the same units as those of the evolutionary distances used to infer the phylogenetic tree. The p-distance method was used to compute evolutionary distances and the Close-Neighbor-Interchange (CNI) algorithm at a search level of 1 was used to determine the strength of the ME tree

**Fig. 3:**
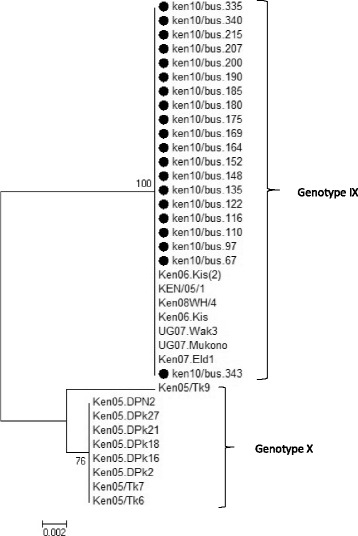
Phylogenetic tree based on the full length *E183L* gene.  Indicates the 20 sequences analyzed in this study that cluster within genotype IX in comparison to 16 reference sequences obtained from Genbank. The evolutionary history was inferred using the Minimum Evolution method after initial utilization of the Neighbor-joining algorithm. The percentage of replicate trees in which the associated taxa clustered together in the bootstrap test (1000 replicates) are shown next to the branches. The tree is drawn to scale, with branch lengths in the same units as those of the evolutionary distances used to infer the phylogenetic tree. The evolutionary distances were computed using the p-distance method and are in the units of the number of base differences per site. The ME tree was searched using the Close-Neighbour-Interchange (CNI) algorithm at a search level of 1

Twenty of the positive samples had their genotype sequences determined. Analysis of the *B646L* gene, revealed that genotype IX was associated with the positive samples. No INDELS were observed in these two polymorphic loci, and the viruses were similar at both loci to virus associated with the 2006 and 2007 ASF outbreaks in Kenya [33]. The phylogenetic tree is illustrated in Figs. 2 and 3 and the Genbank accession numbers for the twenty isolates can be found in Additional file 2.

## Discussion

This study has provided evidence for the presence of ASFV DNA in the blood of visually asymptomatic pigs presenting to slaughter within a period when there were no reported outbreaks in western Kenya. Accurate determination of prevalence in this population is limited mainly by the convenience nature of the sampling (the study design relating to a different pathogen) and the fact that only a sub-set of samples were analysed by both PCR methods. The high number of PCR positive pigs found in this study is interesting, because extensive horizontal household sampling of asymptomatic pigs on farms in the area of the current study between 2011 and 13 (>1000 animals in 600 households; R. Bishop, E. Okoth and C. Onzere, unpublished data), did not reveal PCR positive pigs.

Diagnostic specificity of the USDA ASFV qPCR, using TaqMan EZ reagents, was previously determined within the US National Animal Health Laboratory Network on US populations of domestic and feral swine (Unpublished results). Positive controls performed as expected and no false positives were observed among a negative cohort of EDTA blood samples tested from 895 domestic and 217 feral swine. Diagnostic specificity of the USDA ASFV qPCR was observed to be 100 % using a 95 % confidence interval with lower bound limits of 0.99 and 0.98 for domestic and feral swine, respectively.

ASFV viral DNA has been demonstrated by PCR previously in asymptomatic pigs at slaughter elsewhere in Kenya [40] and neighbouring Uganda [22] as well as in pigs at small holder farms in South-West Kenya [21]. This is not, a consistent finding, with another Ugandan study detecting no viral DNA despite high apparent disease incidence [41].

Genotyping of a sub-set of samples from this study revealed the virus to belong to genotype IX. This genotype appears to have been involved in all outbreaks in Uganda since 1995 and Kenya since 2006, including Ugandan outbreaks in 1995, 2003 [42], 2007 [43], 21 outbreaks between 2011 and February 2013, as well as Kenyan outbreaks in 2006 and 2007 [33]. This genotype was originally thought to be specific to domestic pigs with no evidence of involvement of a sylvatic cycle [42]. It has since been identified in Kenyan warthogs; although, the role of the warthog in recent transmission of the virus to domestic pigs remains unclear [40].

Five months after sampling was completed (January 2011), two outbreaks were reported in western Kenya (Mahiakolo) and neighbouring Nyanza province (Kisumu East) where 163 pigs died, an apparent case fatality rate of 82.5 % according to information submitted to the World Animal Health Information System (WAHIS) [15].

As the last reported outbreak in Kenya prior to sampling was in 2007, and all animals sampled in this study appeared to be asymptomatic based on visual inspection by the research team, the high number of PCR-positive pigs found in this study suggests that virus may have been circulating within the porcine population without clinical signs manifesting. This is surprising as isolates of genotype IX are generally thought to be highly virulent [33]. However, isolates of this genotype have also been detected in apparently asymptomatic pigs at slaughter in Uganda [44]. Isolates of genotype X are generally found to be of lower virulence in experimental infection and have been identified in asymptomatic pigs in Kenya both at slaughter [40] and in the community [21].

Although these East African genotypes are similar overall in genome sequences, there are differences in the multi-copy 360 and 550 family genes encoded at the virus termini. Changes in the copy number and location of these genes appears to be correlated with perceived virulence of the virus isolates in pigs [45].

Antibody detection and virus isolation were not undertaken in this study for several reasons. Antibody detection is notoriously sporadic due to the immunomodulatory effect of the ASFV infection. Antibody detection demonstrates both past and present infections [2] but differentiation of these situations is difficult [46]. It is also apparent that in the case of the East African p72 Genotypes IX and X which are genetically very close [45], the antibody response is often difficult to detect, potentially due to the immunogenetics of the indigenous pig population [40, 43]. Likewise, antibody or low abundance of virus may hinder virus isolation, as animals were apparently asymptomatic at the time of sampling. The process of virus isolation is also difficult and lengthy as there is not a suitable cell line for diagnostic virus isolation of ASFV. ASFV must be propagated in primary porcine macrophages for more than a month or adapted to Vero cells. Neither of these approaches guarantees obtaining a virus isolate. For these reasons, qPCR surveillance represents a fast, sensitive, specific and reliable system for tracking ASFV epidemiology and is currently considered the ‘gold standard’ for ASF genome detection [47].

The data obtained from qPCR does not indicate, however, whether or not the virus present is infectious. While PCR could be used to identify possible routes of virus shedding and transmission, the goal of this study was to determine if ASFV was present within a population of visually asymptomatic pigs in the field and to then characterize the genotype of the virus.

Finding high viral loads within an apparently healthy pig population, is unusual, though not unprecedented, in the field of ASFV virology as ASF is typically associated with 90 to 100 % mortality in pigs [1] [48]. For example, a Ugandan study of over 1300 pigs, established primarily to determine the persistence of viral DNA in asymptomatic animals found only three qPCR positive animals, all of which were directly associated with ASF outbreak events on their farm of origin [41]. High viral DNA loads have, however, been identified in a small number of clinically asymptomatic pigs in Tanzania [23, 48].

A potential explanation for our results is that, although no official outbreak of ASF was reported in Kenya at the time of sampling, we could have detected diseased animals which were being slaughtered illegally. Under-reporting of ASF has been documented in neighbouring Uganda for reasons of distrust of government, poor compensation for destroyed animals and stigmatisation of afflicted farmers [49–51]. Up-to 20 % of farmers in northern Uganda reported “panic sales”, or the quick removal of all animals (sick and healthy) at the onset of an ASF outbreak. They further reported that this activity was taking place clandestinely, often at night [49]. A small study of pork butchers in Busia district (Kenya) suggested that 19 % (3/16) reported buying a pig “after being approached by farmers or after an Africa Swine Fever (ASF) outbreak” [10].

The pigs sampled in this study did, appear on visual examination (by a veterinarian or animal health assistant) to be asymptomatic. It is possible therefore to hypothesis that farmers may not have explicitly sold animals due to clinical ASF, but use alternative cues to decide whether to send animals for slaughter, such as sudden in-appetence. This has been reported in the case of other diseases such as leptospirosis and brucellosis [52, 53]. There is also evidence that farmers may sell their pigs in response to rumours of ASF in their vicinity prior to the detection of sick or dying animals [54]. It would have been very interesting, but unfortunately not recorded in this study, to know which animals were sold due to active approaches by farmers’ to traders or vice versa. The latter scenario being the most common in our study site [10, 55].

In Kenya, official inspection of pigs is required by law at the slaughter facility. The ad-hoc arrangement of the market, in conjunction with an understaffed meat inspectorate in this situation, allows a large proportion of pork to enter the food chain without inspection [25, 56, 57]. In our study area, only 5 % (95 % CI 3–7) of pigs are subjected to ante-mortem inspection, and the slaughter of ‘sick’ animals has been reported in 5 % (95 % CI 3–7) of pig slaughterhouses (Cook et al. In prep.). This poorly regulated slaughter industry provides the opportunity for the unscrupulous slaughter of potentially sick animals. Hence slaughter based surveillance may over-represent disease prevalence and could explain the findings of this study. Similarly, there has been high prevalence of disease detected in market based sampling compared to community-based sampling reported in areas of Uganda endemic for trypanosomiasis in cattle [58]. These scenarios highlight the potential for markets or slaughter facilities to act as sensitive sentinels for disease and central point sampling at such facilities to provide a cost-effective method of disease detection.

Another explanation for the high rates of ASFV detection in this study is that infected pigs may have been brought into the study site for slaughter from neighbouring Uganda where outbreaks had been reported in April, July, August and November of 2010 [59]. The border between these two countries is distinctly porous, and pigs are bought and sold across the international border [10, 54]. Studies of farmers, butchers and pig traders in this area, suggest that the majority of pigs originate within 1–20 km [10], with the vast majority travelling <5 km although this pattern may be disrupted in cases of ‘panic selling’ [54]. This hypothesis might explain some of the PCR-positive pigs identified in the study, the spatial distribution of the positive samples, throughout the study site, does not support the theory of extensive cross-boundary incursion in the current situation.

The system of procuring pigs for the slaughterhouses and slaughter slabs in this study are very informal. For the most part, a ‘scout’ is sent out from the slaughterhouse to the community of villages around it to convince farmers with appropriately aged pigs to sell them for that day’s slaughter. As such, all pigs slaughtered in a given facility are drawn from the farms in the immediate vicinity, and the population of pigs at slaughter differs only from those in the community by age - pigs in the community represent all age groups, those sold for slaughter represent pigs aged approximately 9–12 months old. The pork meat from these facilities is sold to local butcheries (usually owned by the same people as the slaughterhouse) for small scale sale to the local community. All pigs in this study were reported to have originated from within Kenya (See Additional file 1), and as the majority of pigs are brought to slaughter by bicycle or foot [10], we believe that only pigs being slaughtered in close proximity to the border might have originated from Uganda. We are confident that the pigs in this study represent pigs of slaughter age drawn from the community surrounding the slaughterhouses.

Our results suggest, that there is ASFV circulating within the pig population without clinical signs being detected. Several mechanisms for the persistence of virus in asymptomatic pig populations have been suggested, such as chronic or sub-clinical infections or low virulence isolates, but these do not provide sufficient explanation for the situation described in this manuscript. As mentioned previously, chronic infections are not believed to occur on the African continent, Ct values would have been expected in the range of 30–40 [18] and symptoms such as emaciation and pathologic lesions such as skin necrosis, arthritis, fibrinous pleuritis, peri-carditis, pleural adhesions and necrotic pneumonia should have been observed in the animals [2].

Genetic resistance to ASFV infections has been proposed previously but the heritability has not been proven [6]. Interestingly genomic analysis of 117 pigs included in the current study revealed that animals testing negative for ASFV on conventional PCR at ILRI (*n* = 65) had significantly (*P* = <0.0001) higher indigenous ancestry, (54 % and above) compared to those testing positive (*n* =52) (Mujibi, Okoth, Onzere, Bishop, Fevre, Thomas, Plastow, Rothschild*, In prep*).

The presence of infected pigs at abattoirs and in turn the dispersal of potentially infective meat to butcheries across the study site is of particular importance to the control of ASF. Previous studies have identified the movement of infected pigs to an abattoir and the movement of infected pork products as being major risk factor for outbreaks, which appear to be distinct from the sylvatic cycle [3, 60]. Unregulated slaughter increases the risk of infected pork meat being sold, and infective material being transported into un-exposed areas through contact with fomites (including bicycles, vehicles, slaughter staff clothing, and machete), or becoming pig swill. These mechanisms could lead to transmission of the virus [4, 61] and may have been instrumental for initiation of the outbreak which occurred soon after our sampling period ended.

The discordance between the large number of ASFV positive pigs in this study in comparison to a community based study in the same area suggests that slaughter slab data provides an overestimate of the overall prevalence of ASFV in the local population. This is potentially promoted by anthropogenic factors, such as panic selling of potentially diseased pigs, and implies that slaughter slab data alone cannot be used to estimate true prevalence with clear implications for ASFV transmission dynamics modelling.

## Conclusion

The detection of ASFV viral DNA in a relatively high number of pigs delivered for slaughter during a period with no reported outbreaks provides support for two hypotheses, which are not mutually exclusive: (1) that virus prevalence may be over-estimated by slaughter-slab sampling, relative to that prevailing in the wider pig population; (2) that sub-clinical, chronically infected or recovered pigs may be responsible for persistence of the virus in endemic areas.

## Additional files

Additional file 1: Results of conventional and real-time PCR. Contains sample ID and sampling location with the results from the conventional PCR assay and the real-time PCR assays using the TaqMan® EZ-RT-PCR Kit and the Life Technologies Path-ID Multiplex One Step Kit. (XLSX 43 kb) Additional file 2: GenBank Accession Numbers. GenBank (http://www.ncbi.nlm.nih.gov/genbank/) Accession numbers for twenty ASFV isolates for which genotypic analysis was performed. (XLSX 11 kb)
